# The Use of Telemedicine in Medical Education and Patient Care

**DOI:** 10.7759/cureus.37766

**Published:** 2023-04-18

**Authors:** Lana Shawwa

**Affiliations:** 1 Medical Education, King Abdul Aziz University, Jeddah, SAU

**Keywords:** remote teaching, online, education, medical education, telemedicine

## Abstract

The COVID-19 pandemic has accelerated and expanded the adoption of telemedicine globally. This allowed telemedicine to engage medical students in patient care and ensured continuity of care for vulnerable patients. In this review, the history of telemedicine and some of its applications in medical education were reviewed. Furthermore, we also shed light on how to incorporate telemedicine into several curricula and the strategies used to include it. The article also explored how to evaluate telemedicine and the major facilitators and barriers any medical and educational institution must address when using telemedicine. At the end of the review, we explored the future promises telemedicine has for medical education.

## Introduction and background

Telemedicine was defined by the World Health Organization (WHO) as “the delivery of healthcare services, where distance is a critical factor, by all healthcare professionals using information and communication technologies for the exchange of valid information for diagnosis, treatment, and prevention of disease and injuries, research and evaluation, and for the continuing education of health care providers, all in the interests of advancing the health of individuals and their communities” [1].

History of telemedicine

Telemedicine has a long and interesting history that dates back to the 19th century. In the 1860s, during the American Civil War, the telegraph was used to inform medical teams about injured soldiers [2]. After William Einthoven created his first ECG in the early 20th century, information was shared between his lab and academic institutions [3]. Later the telegraph was replaced by radio and telephone which was used primarily for providing ship personnel with healthcare advices [4]. Between 1940 and 1970, there were several successful transmissions of clinical data, both over short distances and across continents [5]. After its invention, the television was also used in medical education. In 1944, Johns Hopkins Hospital broadcasted an operation through the aid of closed-circuit television to a large number of surgeons [5]. Between 1970 and 2000 rapid and substantial revolutions in connectivity and devices led to the initiation of contemporary telemedicine. The phrase “telemedicine” was first proposed by Murphy and Bird [6] in 1974 after they successfully conducted 1000 patient encounters between the medical station of Logan International Airport and the emergency ward of Massachusetts General Hospital [6]. The biggest advancement in connectivity, the availability of the internet to the general population in addition to rapid advancement in technology facilitated telemedicine development [5]. By incorporating virtual hospitals into medical care, telemedicine has been involved with a diverse scope of services including and not limited to neonatology, fetal care, neonatal resuscitation, remote rounding, tele-specialty consultations, and parental education [5]. In 2020 a study on the US telemedicine market stated that at that time more than 75% of United States (US) hospitals had some form of telemedicine program [7]. Nowadays healthcare providers, using live chats and video calling, are providing healthcare directly to patients in their households.

## Review

Approaches to telemedicine

There are two common approaches to telemedicine. The first is communication, distant monitoring, and access to data. Through this approach, health professionals can access patients’ files, read and interpret test results, and monitor vital signs from anywhere [8]. Some of the telemedicine gadgets might include video conferencing software, wearable health monitoring devices, digital stethoscopes, telemedicine carts, and mobile health apps [9]. The second approach is remote clinical services where health professionals can perform a wide range of clinical tasks. Those tasks can be as simple as prescribing medications, to more advanced services as conducting surgery, via robotic instruments [10]. Among the first disciplines that adopted telemedicine was radiology. The introduction of Picture Archiving and Communication Systems (PACS) enabled clinicians to store, access, and interpret radiographs without the necessity for expensive films and with high levels of precision [11].

Telemedicine and medical education

The current experiences associated with the incorporation of telemedicine in medical education were explored. Waseh and Dicker emphasized how undergraduate medical education preclinical years can be central to telemedicine training and exposure [12]. During the preclinical years, medical students are primarily focused on learning the foundational knowledge of medicine through classroom lectures, textbook readings, and hands-on laboratory exercises. However, with the advent of telemedicine, medical students can now access a wealth of information and resources through the use of technologies such as video conferencing which can aid in conducting thematic discussions and presentations [13].

The majority of telemedicine integration projects were applied during the clinical years. For instance, the University of Nebraska has integrated telemedicine into its doctoring stream, whereas the University of Maryland teaches fundamental telemedicine concepts during lecture time. In order to allow students to practice their clinical abilities while receiving formative input, Oregon Health and Sciences University has integrated telemedicine into their objective structured clinical assessments [14].

Iancu, Kemp, and Alam in 2020 [15] suggested that by incorporating telemedicine into their curricula, medical schools can augment their students’ education and teach them core competencies for patient care, thus providing much-needed medical education as well as some clinical experience. Additionally, they noted that by incorporating telemedicine into undergraduate medical school, all aspiring doctors will have access to this form of training, which can significantly reduce healthcare costs and increase patient access to care [15]. Medical schools can teach their medical students clinical care through existing telemedicine hospital systems rather than recreating new technology platforms [15].

A student-centered telemedicine program was provided to 149 third-year medical students by Jonas et al. [16]. First, a presentation was given to students on telemedicine's history, uses, and ethics. After the lectures, interactive telemedicine teaching sessions with simulated patient consultations and hands-on training with telemedicine tools were conducted. Ten percent average improvement between pre- and post-test assessment scores was found [16].

Costich et al. [17] explained how telemedicine can be incorporated into a medical curriculum. In their experience, the curriculum aims and objectives were created through literature analysis and consulting with telemedicine subject matter experts. Next came the development of instructional materials through utilizing institutional telemedicine training resources and real patient situations. The fundamentals of telemedicine were reviewed, including how to sign up for, set up, record, and bill for a video visit as well as how to carry out crucial elements of the physical examination [17].

Telemedicine can be a powerful tool for teaching leadership and communication skills in the healthcare setting. Leadership and communication are essential components of effective healthcare delivery, and telemedicine provides a unique opportunity to train healthcare providers in these areas. In their work, Liu et al. [18] used a telemedicine communication skills training platform that was part of the undergraduate curriculum. Their aim was to provide clinical communication skills training that integrates nonverbal behavior assessment for medical students [18]. They provided their students with additional practice opportunities with standardized patients so students could review their nonverbal communication behaviors. They compared the efficacy of face-to-face and remote consultation and discovered that the students' medical communication abilities were enhanced by remote consultation. This could be explained by the enhancement of the existing telehealth systems by providing students with multiple kinds of feedback [18].

Telemedicine was also utilized to deliver ethics courses to medical students [19]. It was created by two medical school clinicians from different continents. Second-year medical students had the opportunity to examine a number of aspects of quality of life in dialysis patients during the lecture, "Exploring the Patient Experience Via Telemedicine: Dialysis and End-Stage Renal Disease" [19].

Embedding telemedicine into the curriculum of medical schools can help students better comprehend complicated ethical, regulatory, and legal challenges in addition to introducing them to pertinent technologies [20].

Strategies to teach telemedicine

Lectures and patient interactions were two widely utilized teaching techniques in telemedicine. As an initial step, lectures were used to convey the fundamental ideas behind telemedicine whether teaching an ethics course or communication skills [20-22]. Other methods focused on patient encounters through using video-based communication [18,19]. To a lesser degree telemedicine was taught through simulation-based education [23], workshops [24], reflection, and reflective writing [19,25]. The length of the sessions that taught telemedicine ranged from one hour [20] to 13 weeks [19]. Naturally, the competencies needed for telemedicine encounters differ from those needed for conventional encounters. Without specialized training in these special abilities, doctors frequently omit physical exam techniques or appear distant via a computer screen, resulting in lower-quality care for patients [24]. The fundamental definitions of virtual medicine, pertinent ethical and legal guidelines, maximizing patient safety, and physical exam techniques specific to the virtual world, including web etiquette, should all be understood by students [24].

Preparing students and faculty for telemedicine

In their article, Iancu et al. [25] offered advice on how to prepare medical students for telemedicine visits at a departmental or institutional level. They stressed the necessity to form a task force for telemedicine that comprises a wide range of diverse stakeholders who play a variety of roles in clinical care and medical education. Effective integration of telemedicine in medical education systems also required encouraging the involvement of both the educational and clinical leadership. Understanding how telemedicine is used differently in various departments and specializations is crucial for integrating students into the field. In order to create a set of realistic group goals, objectives, and expectations, medical educators should work to better understand this distinct utilization [25]. Other factors should be taken into consideration such as knowing the institution's requirements for telemedicine technology and software, developing a functional workflow, and teaching telemedicine principles and skills to both faculty members and students [25]. It is also important to discuss and address some of the disadvantages associated with telemedicine. García-Gutiérrez et al. [26] found that students perceived the disadvantages of telemedicine more often and had a high self-reported perception of the shortcomings of telemedicine, especially with the efficiency of care, patient security, and the possibility of medical misconduct compared to medical doctors [26].

Facilitators and barriers of telemedicine

One of the primary facilitators of telemedicine is the increasing availability and affordability of technology advancements in telecommunication technologies, such as high-speed internet connections and video conferencing software. With the widespread availability of smartphones, tablets, and computers, patients are now able to easily access healthcare services from their own homes [27-29]. User-friendliness was also found as a major facilitator in several studies, and it was linked to high satisfaction in patients and health personnel, system efficiency, and acceptability [27,30,31].

Another facilitator of telemedicine is the changing healthcare landscape. With the increasing focus on value-based care, healthcare providers are looking for ways to provide high-quality care while reducing costs. Telemedicine offers a cost-effective solution that can help to reduce the cost of delivering care while improving patient outcomes and satisfaction [32].

Regulatory changes have also facilitated the growth of telemedicine. In recent years, many states have passed laws and regulations that support the use of telemedicine. Some states in the US have enacted laws that require insurers to cover telemedicine services, while other states have relaxed licensing requirements for healthcare providers who deliver care remotely [33].

Despite the potential facilitators of telemedicine, there are several barriers that hinder its widespread adoption and utilization. One of the major barriers to telemedicine is the lack of reimbursement policies. In many countries, insurance does not provide coverage for telemedicine services and is not reimbursed by government programs. As a result, healthcare providers may be reluctant to adopt telemedicine due to concerns about financial sustainability. Additionally, patients may be hesitant to use telemedicine if they are required to pay out of pocket for the services. Without proper reimbursement policies, the growth of telemedicine may be limited [34,35].

Another significant barrier is the digital divide. While the availability of high-speed internet and mobile devices has increased in recent years, there are still many areas where access to these technologies is limited. Rural and low-income areas are particularly affected by the digital divide, which may limit the ability of patients to access telemedicine services. Additionally, older adults and those with limited technology skills may have difficulty using telemedicine platforms. Bridging the digital divide is crucial for ensuring equitable access to healthcare services through telemedicine [36,37].

Privacy and security concerns are also major barriers to telemedicine. Healthcare providers must ensure that patient information is protected and secure when using telemedicine platforms [38]. However, there are several challenges in maintaining privacy and security in telemedicine. For example, patients may use unsecured internet connections or devices, which may make them vulnerable to cyber-attacks [39]. Addressing privacy and security concerns is essential for building trust among patients and healthcare providers in telemedicine [40].

Another challenge is the lack of standardized regulations and guidelines across different countries and regions for telemedicine which may create confusion and uncertainty for healthcare providers and patients [41]. Additionally, the lack of standardization may hinder interoperability between different telemedicine platforms and systems, making it difficult for healthcare providers to exchange information and coordinate care [42].

Finally, there are several cultural and social barriers to telemedicine where for example some patients may prefer face-to-face interactions with healthcare providers, which may limit their willingness to use telemedicine [43]. Addressing cultural and social barriers requires a comprehensive approach that involves education, outreach, and community engagement.

Evaluation of telemedicine initiatives

Many stakeholders such as health professionals, technology manufacturers, and educational institutions to name a few are interested in the future of telemedicine. Although research on evaluating telemedicine is expanding there is still a need to conduct well-designed studies, devise strategies, and secure resources to provide decision-makers with reliable information on the benefits, risks, and costs of specific telemedicine applications [44].

Both the process of development and investments in telemedicine necessitates gathering data to support the benefits and expected profits from the viewpoints of the patient and the health service provider. Analyzing all available data regarding the use of telemedicine, its availability, accessibility, cost-effectiveness, and affordability can help in evaluating its status. This evaluation will help in recognizing requirements, examining the existing possibilities, and understanding the role of telemedicine in refining healthcare [45].

Telemedicine evaluation can be attained using a variety of theoretical frameworks or different evaluation models. To select the proper approach, one should consider several issues. Those issues involve realizing project goals; defining the time, budget, and energy needed; choosing the proper method based on the predetermined metrics and criteria; choosing a user-friendly evaluation method; and using validated methods [46].

The instruments that are most frequently used to gauge customer satisfaction in the field of telemedicine are questionnaires and interviews [47]. Hajesmaeel‑Gohari et al. [47] reviewed the most commonly used questionnaires that explored the characteristics of telemedicine and were used for assessing telemedicine services. They recommended to use specifically designed questionnaires or create new ones that have fewer elements and more comprehensive questions. They also recommend that any questionnaire that evaluates a telemedicine initiative has to pay close attention to user needs and acceptance, implementation process, usability, and users’ satisfaction [48].

In his book “Telemedicine: A guide to assessing telecommunications for health care”, Field [44] suggested approaches that explored the logic and practicability of any telemedicine intervention. He also stated that conducting assessment studies that present the perceived value and operational feasibility was needed. Another approach mentioned was advising to conduct clinical trials that thoroughly collect and analyze data on the telemedicine intervention's effect [44].

In 2012 Kidholm [45] proposed the Model for Assessment of Telemedicine (MAST) as a multidisciplinary framework that measures both qualities of care and effectiveness. MAST evaluates the social, medical, economic, and ethical aspects of telemedicine [45]. It consists of three steps, and it starts by assessing the maturity of the telemedicine technology and the institute using it. This is followed by a multidisciplinary assessment, and it includes several domains. The domains contain the identification of the health problem and features of the application; clinical effectiveness; patient perspectives; safety; organizational aspects; economic aspects and socio-cultural, ethical, and legal aspects. Finally, the last step involves an assessment of the transferability of the results stated in studies addressing the preceding steps [46].

In 2017 Al Dossary et al. [49] proposed a planning framework for evaluating telemedicine services which were based on needs assessment. The framework involves two phases. The first phase includes an assessment of availability and stated needs; convenience, perception, and affordability. Phase two involves ranking the demands of needed health services, taking into consideration the limitations of supply, and the application of a suitable telemedicine service that echoes and addresses the needs of the public [49].

Poultney [50] summarized the challenges for evaluating telemedicine services with three issues: the lack of a universally accepted standard, the variable weaknesses of existing methodologies in terms of suitability, and partial exposure of evaluation focus on clinical-benefit and cost-benefit.

Future of telemedicine

Telemedicine has emerged as a valuable tool for delivering healthcare remotely, particularly during the COVID-19 pandemic. It allowed patients to connect with healthcare providers through videoconferencing, phone calls, or messaging platforms, enabling access to medical care from the comfort of their homes. Daily, many patients and clinicians embrace telemedicine thus shaping the future of health care [51]. With the development of wearable devices and electronic health records, telemedicine is growing stronger and providing comprehensive and coordinated care [52].

Currently, the improved regulatory and reimbursement frameworks aided telemedicine to ensure that patients have access to high-quality and affordable telemedicine services. For example, in the US, the reduction of licensing restrictions and insurance coverage by Medicare helped telemedicine to be in high demand [51].

Advancements in technology, such as the Internet of Things (IoT) [53], 5G networks, artificial intelligence (AI) [54], and big data analytics [55] improved the capabilities and effectiveness of telemedicine. Many countries are currently using digital technology-based platforms. Four modes of such platforms are one-to-many, many-to-one, consultations, and practical modes [56].

In 2019 Tipton [57] presented nine steps of transformational change and currently, telemedicine has passed the tolerance stage and moving into the acceptance stage. This means that telemedicine as a transformational change has been routinely used and its users feel familiar with the technology. Not only that the users are feeling that both face-to-face and remote care are equal. This poses a challenge and restricts in-person visits [56].

When addressing the future of telemedicine, one must also mention the significant impact telemedicine will have on elderly care, especially in hospice and nursing homes. Telemedicine not only will provide access to healthcare for elderly individuals who may have limited mobility or face difficulty traveling to appointments. Through telemedicine, health professionals can monitor several health parameters and vital signs, such as blood pressure, heart rate, and glucose levels. The elderly knowing that all their parameters are monitored can have an increased feeling of safety and reassurance [58].

Another telemedicine future implication is that it will reshape the dynamic of the doctor-patient relationship. Virtual appointments may feel less personal than in-person visits, and healthcare professionals may need to find ways to establish rapport and build trust with patients through digital means [59]. Telemedicine can also bring in greater patient autonomy where patients may have more control over their healthcare, as they are able to access medical advice and treatment without having to rely on their doctor's schedule or availability. Patients may also have access to a wider range of healthcare services, as they are not limited by geographic location [60].

COVID-19 sped up the progress of telemedicine systems, in their research Busso et al. found that the number of calls increased by 230%, and the number of first-time callers increased by 198%. They also found that the increase in the use of telemedicine slightly diminished after mobility restrictions lessened, but not enough to undo the increase [61]. All this should emphasize that more education, greater outreach, and infrastructure support are still needed for patients in underrepresented and marginalized areas [62].

## Conclusions

Telemedicine refers to the practice of providing medical care and services remotely using technology, such as video conferencing, phone calls, messaging, and other forms of digital communication. It allows healthcare providers to consult with and treat patients who are in different locations, often without requiring them to leave their homes or travel to a healthcare facility. Telemedicine has become increasingly popular in recent years, as advances in technology have made it easier and more affordable to connect patients with healthcare providers remotely. Moreover, it can be used to diagnose and treat a wide range of medical conditions, including chronic diseases, mental health issues, and acute illnesses.

One of the benefits of telemedicine is that it can improve access to healthcare for people who live in rural or remote areas, or who have difficulty traveling to healthcare facilities. It can also save time and money for both patients and healthcare providers, as it eliminates the need for in-person visits and can often be done more quickly and efficiently.

Though telemedicine faces many challenges such as the lack of funds, lack of experience, infrastructure, etc., it still provides safe, affordable, and convenient medical services and proved to be highly effective during the COVID-19 pandemic. Future work is still needed on how to improve the use of telemedicine, the ethical and legal implications, and patient safety.

Telemedicine is also a vital tool for medical education as it allows medical students to gain valuable experience in diagnosing and treating patients remotely. This technology allows students to participate in virtual consultations, observe procedures and surgeries, and access medical records and patient data remotely. It can also provide medical students with opportunities to practice their communication and critical thinking skills in a virtual environment, which can improve their clinical decision-making abilities. Additionally, telemedicine can aid health institutes overcome challenges related to limited resources and access to clinical experiences by providing students with opportunities to interact with patients and healthcare providers from different sites and specialties. Overall, telemedicine has the potential to enhance the quality and accessibility of medical education, ultimately leading to better healthcare outcomes for patients.
